# Genetically Modified MSCs for Targeted Regeneration: Balancing Efficacy, Biosafety, and GMP Standardization

**DOI:** 10.3390/cells15151406

**Published:** 2026-08-03

**Authors:** Kristina V. Kitaeva, Ivan Y. Filin, Albert A. Rizvanov, Shahlo Turdikulova, Mirakbar Yakubov, Oksana Charishnikova, Valeriya V. Solovyeva

**Affiliations:** 1 Institute of Fundamental Medicine and Biology, Kazan Federal University, 420008 Kazan, Russia; krvkitaeva@kpfu.ru (K.V.K.); ivyfilin@kpfu.ru (I.Y.F.); vavsoloveva@kpfu.ru (V.V.S.); 2 Division of Medical and Biological Sciences, Tatarstan Academy of Sciences, 420111 Kazan, Russia; 3 Center for Advanced Technologies, Academy of Sciences of the Republic of Uzbekistan, Tashkent 100174, Uzbekistan; shahlo.ut@gmail.com (S.T.); mirakbardan@yahoo.com (M.Y.); charaoxa@gmail.com (O.C.)

**Keywords:** mesenchymal stromal cells, genetically modified MSCs, lentiviral vectors, genome editing, safe-harbor integration, hypoimmunogenic MSCs, GMP

## Abstract

Mesenchymal stromal cells (MSCs) are a versatile platform for regenerative medicine and gene delivery because they combine multipotency, immunoregulatory activity, and injury-directed trafficking. Translation is nevertheless limited by donor- and tissue-dependent heterogeneity, variable biodistribution, and engineering-related risks. This review evaluates genetically modified MSCs as medicinal products rather than as a general MSC class. We compare self-inactivating lentiviral (SIN-LV) transduction, which provides efficient and durable expression and has limited early clinical experience, with targeted genome editing, which can define the integration locus and copy number but remains constrained by variable precise knock-in efficiency, off-target and double-strand-break-associated effects, manufacturing cost, and the absence of long-term clinical safety data. We integrate preclinical and clinical evidence with GMP-compatible manufacturing, potency testing, genomic surveillance, and release criteria. Particular attention is given to safe-harbor integration and B2M/CIITA-based hypoimmunogenic designs as strategies to reduce engineering-related batch variability and HLA-dependent donor variability. Together, these developments support a transition from empirically optimized MSC preparations toward molecularly defined cellular medicines with predefined genotype, expression, potency, and safety attributes.

## 1. Introduction

Mesenchymal stromal cells (MSCs) are multipotent stromal cells that can differentiate into osteogenic, chondrogenic, and adipogenic lineages. First characterized in bone marrow by Friedenstein and colleagues, MSCs can also be isolated from adipose tissue and perinatal tissues, including the placenta and umbilical cord. Their therapeutic appeal reflects a combination of paracrine activity, immunoregulation, and migration toward injured or inflamed tissues [1,2].

Unmodified MSCs have been evaluated in inflammatory, orthopedic, cardiovascular, and pulmonary disorders, but efficacy remains variable despite an overall favorable safety profile [3]. Genetic engineering can convert MSCs from variably active cell preparations into living delivery systems for defined proteins, RNAs, receptors, or oncolytic payloads. Lentiviral vectors remain the dominant platform for durable expression, whereas targeted nucleases and newer base- and prime-editing systems increasingly permit sequence-defined changes. This technological expansion occurs within a broader cell- and gene-therapy pipeline that included more than 3700 clinical and preclinical programs in 2023 [4].

Each engineering modality introduces a distinct risk profile. Lentiviral transduction can produce variable vector copy number and semirandom integration, with a residual risk of insertional mutagenesis. Targeted editing can reduce integration-site uncertainty, but primary MSCs are difficult to edit precisely and may acquire off-target mutations, large on-target rearrangements, or manufacturing-related stress. Therefore, identity, genomic integrity, potency, and biodistribution must be evaluated together rather than as independent attributes [2].

Accordingly, the field is moving from empirically optimized MSC preparations—selected through donor screening, tissue sourcing, culture conditions, and priming—toward molecularly defined cellular medicines in which genotype, integration site and copy number, expression circuit, potency, and safety attributes are specified in advance. Unlike broad reviews of MSC biology, this review examines genetically modified MSCs as translational medicinal products through three connected dimensions: therapeutic efficacy, biosafety of stable engineering, and GMP-compatible manufacturing and quality control. This framework also allows for the direct comparison of mature SIN-LV approaches with emerging targeted genome engineering.

## 2. Heterogeneity of Mesenchymal Stromal Cells and Standardization of Their Clinical Application

MSC heterogeneity arises from differences in donor characteristics, tissue source, manufacturing protocols, and cryopreservation, all of which influence therapeutic potency and product consistency [5,6,7,8,9,10,11,12,13,14,15,16,17,18,19,20,21,22,23,24,25,26,27,28,29,30,31,32,33,34,35,36]. This biological variability remains one of the principal challenges for the development of standardized MSC-based therapies. Both donor- and manufacturing-related variables contribute to functional diversity. Tissue source and donor characteristics are major contributors to this variability. MSCs isolated from bone marrow, adipose tissue, and perinatal tissues differ in cell yield, proliferative capacity, differentiation potential, and immunomodulatory properties, while donor age and sex further affect regenerative function [7,8,9,10,11,12,13,14,15,16,17,18,19,20,21,22,23,24]. Additional variability is introduced during manufacturing through differences in cell isolation, culture conditions, serum supplementation, oxygen tension, and cryopreservation [25,26,27,28,29,30,31,32,33,34,35,36]. Representative characteristics of the principal MSC sources are summarized in Table 1.

Despite increasing efforts to standardize manufacturing, substantial biological heterogeneity persists between donors and even within individual MSC preparations. Consequently, the development of genetically engineered MSC products is increasingly viewed not only as a means of introducing new therapeutic functions but also as an approach to improve product consistency and reduce donor-dependent variability.

### 2.1. Biological Properties Relevant for Therapeutic Engineering

#### 2.1.1. Immunomodulatory Properties and Their Enhancement

MSCs exhibit relatively low immunogenicity owing to the limited expression of HLA-DR and co-stimulatory molecules, although inflammatory stimulation can enhance antigen presentation [37,38,39,40]. Beyond this intrinsic phenotype, MSCs actively regulate both innate and adaptive immune responses by suppressing T- and B-cell activation, modulating dendritic cells and macrophages, promoting regulatory immune populations, and secreting multiple immunoregulatory mediators [41,42,43,44,45,46,47,48,49]. These biological properties have enabled broad clinical evaluation of allogeneic MSC products across immune-mediated and degenerative diseases [50,51,52,53,54,55]. Nevertheless, repeated administration may still induce donor-specific immune responses [56,57,58], highlighting the need for strategies that enhance immunoregulatory activity while reducing residual immunogenicity.

MSC immunomodulatory activity can be enhanced using either genetic engineering or transient priming approaches. Stable genetic modification enables sustained expression of therapeutic molecules such as IL-10, IDO1, or PD-L1, whereas priming temporarily activates endogenous immunoregulatory pathways without altering the genome [59,60,61,62]. Although both approaches improve MSC function, they differ in durability, manufacturing complexity, and regulatory considerations.

#### 2.1.2. Homing and Its Enhancement

Efficient homing remains one of the principal limitations of MSC therapy because only a small fraction of systemically administered cells reach the target tissue [63,64,65,66,67,68,69,70,71,72,73,74,75,76,77,78,79]. Migration efficiency depends on chemokine receptors, adhesion molecules, tissue source, donor characteristics, and culture conditions, whereas pulmonary entrapment and poor post-transplant survival further reduce therapeutic cell delivery [63,64,65,66,67,68,69,70,71,72,73,74,75]. Consequently, numerous genetic and non-genetic engineering strategies have been developed to improve MSC trafficking and tissue retention, as discussed in the following sections.

#### 2.1.3. Enhancement by Conditioning

Biophysical and metabolic conditioning can also reshape the secretome. Fluid shear stress activates NF-κB-dependent COX-2 and IDO1 pathways and increases PGE2/IDO1-mediated suppression without reducing viability in the reported model [62]. Hypoxic preconditioning (typically 2–5% O_2_) activates HIF-1α and can improve stress resistance, migration, immunoregulation, and extracellular-vesicle output [74]. A short post-thaw acclimation period can restore functions that are transiently impaired immediately after cryopreservation [34]. Because priming effects are temporary and context-dependent, GMP protocols should define the stimulus, dose, duration, washout, recovery interval, and a mechanism-linked potency assay rather than treat priming as an interchangeable culture step.

## 3. Quality Control and Biosafety During Scale-Up

An MSC-based medicinal product must be controlled from tissue procurement through isolation, expansion, genetic modification, cell banking, formulation, cryopreservation, and administration. Unlike a conventional sterile drug, the final living product cannot be terminally sterilized and may have limited stability and lot size. Source variability and process-induced phenotypic drift therefore have to be managed through predefined critical process parameters and critical quality attributes.

Clinical doses commonly require approximately 1–8 × 10^6^ cells/kg, depending on the indication [80], making ex vivo expansion and scale-up unavoidable. Prolonged culture can reduce proliferation and multipotency, induce senescence, accumulate DNA damage, and select cytogenetically abnormal clones [81,82]. A release strategy should therefore combine identity and viability, MSC immunophenotype, sterility, endotoxin and mycoplasma testing, karyotypic or genomic stability, and a mechanism-linked potency assay [81,83,84]. For engineered products, these conventional attributes must be supplemented with measurements that are specific to the modification platform.

### 3.1. Minimal Criteria for Defining Multipotent Mesenchymal Stromal Cells

The International Society for Cellular Therapy proposed three minimal criteria for cultured MSCs. These criteria establish identity but do not by themselves demonstrate therapeutic potency or engineering quality:

Plastic adherence under standard culture conditions.

Expression of CD105, CD73, and CD90 by at least 95% of cells, with no more than 2% positive for CD45, CD34, CD14 or CD11b, CD79α or CD19, and HLA-DR.

Capacity for osteogenic, adipogenic, and chondrogenic differentiation under standard in vitro conditions [85].

For genetically modified MSCs, fulfillment of the ISCT criteria should be confirmed after engineering, enrichment or cloning, expansion, and cryopreservation. The comparison with the unmodified parental bank is particularly important because editing and selection can preserve surface markers while altering secretory potency, senescence, or biodistribution.

### 3.2. Genomic Stability and Identity Testing

Expansion and genetic manipulation can generate or select chromosomal abnormalities. G-banding, fluorescence in situ hybridization (FISH), and spectral karyotyping provide complementary information on numerical and structural alterations, with spectral methods detecting abnormalities that may be missed by conventional banding [86]. No single assay is sufficient for all product risks, and the sampling time point must reflect the final manufacturing history.

Some cultures show stabilization or loss of abnormal clones at later passages, possibly because those clones proliferate poorly; this observation should not be interpreted as evidence that extended culture is intrinsically safe [87]. Passage number, population doublings, donor, culture duration, and any enrichment bottleneck should be recorded when genomic-stability results are interpreted.

Cytogenetic methods have limited sensitivity for small variants and low-frequency subclones. Targeted or genome-wide sequencing can increase resolution [88], although routine use is constrained by cost, validation requirements, data interpretation, and the absence of universally accepted thresholds [89]. Proteomic profiling may complement functional characterization but is not a substitute for direct genomic analysis [90].

Short tandem repeat (STR) profiling is the standard method for confirming cell-line and bank identity [91]. The STR profiles of the master cell bank, working cell bank, and final product should be concordant at validated loci. For pooled products, donor-specific traceability requires an explicitly validated strategy because a simple consensus STR profile may be insufficient.

**Figure 1:**
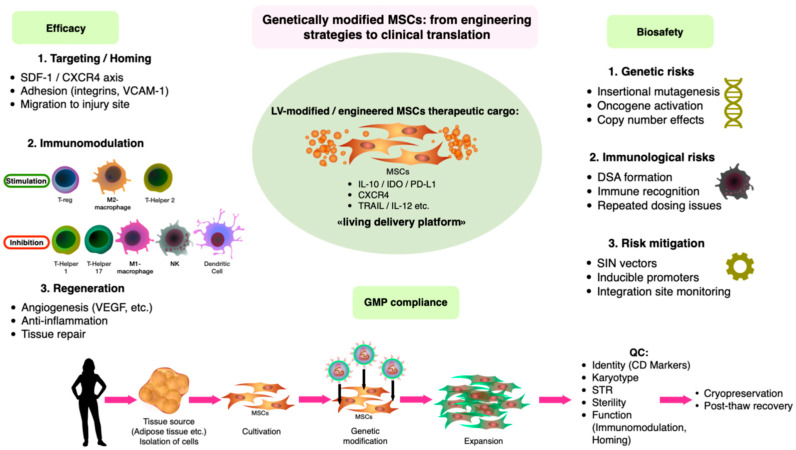
Manufacturing and translational workflow for genetically modified mesenchymal stromal cells (MSCs). The illustrated engineering example centers on lentiviral modification of MSCs as living delivery vehicles for cargos such as IL-10, CXCR4, or TRAIL. Efficacy-related attributes include SDF-1/CXCR4-dependent trafficking, immunomodulation, angiogenesis, and tissue repair. Biosafety controls include vector design, regulated expression, genomic surveillance, and monitoring of immunological and biodistribution risks. The GMP workflow comprises source-specific isolation, engineering, scale-up, identity and potency testing, genomic-stability assessment, cryopreservation, post-thaw recovery, and clinical administration. Targeted editing follows the same downstream framework but requires additional on-target, off-target, copy-number, and residual-editor assays.

Telomerase activity, measured, for example, by the telomeric repeat amplification protocol (TRAP), can support assessment of replicative state and unexpected immortalization [92,93,94,95], but it should be interpreted together with growth kinetics, senescence markers, karyotype, and tumorigenicity. Figure 1 summarizes the manufacturing workflow for genetically modified MSCs from tissue collection and cell banking through engineering, expansion, quality control, cryopreservation, post-thaw recovery, and administration.

### 3.3. Potency and Purity Assays

Potency testing is challenging because MSC activity is mediated by overlapping mechanisms. EMA guidance requires the assay to reflect the intended mechanism of action and to discriminate subpotent lots [EMA/CAT/80183/2014; EMA/CAT/571134/2009]. A matrix of orthogonal assays may therefore be more informative than one marker, for example, a cytokine-licensed T-cell-suppression assay combined with quantification of IDO1 or PGE2, or a migration assay combined with CXCR4 expression for a homing-focused product.

Many immunoregulatory mediators are inducible rather than constitutively secreted [96,97]. Donor sex can also affect assay output: female-donor AD-MSCs produced approximately twice as much IDO1, PGE2, and IL-1RA as male-donor cells under the reported conditions [98]. Potency assays must therefore specify the activation stimulus and should be qualified against a biological response, not used as unconstrained measurements of basal secretory activity. Biodistribution remains a separate in vivo attribute and should not be inferred from an in vitro cytokine panel.

Purity testing includes residual hematopoietic or non-MSC populations, microbial contaminants, endotoxin, mycoplasma, and process-related impurities. For LV-modified MSCs, additional release or characterization assays include vector copy number, integration-site distribution when justified by risk, residual vector components, and replication-competent lentivirus. For genome-edited MSCs, the analogous panel includes on-target allele frequency and zygosity, intended and unintended donor integration, off-target variants, large on-target rearrangements, residual nuclease or guide RNA, and the clonal composition of the final population.

A molecularly defined product therefore requires more than a sequence-confirmed edit. The genotype must be linked to stable expression, preserved MSC identity, a mechanism-relevant potency result, acceptable genomic integrity, and a controlled manufacturing history. Safe-harbor integration can reduce batch-to-batch variability caused by random insertion and variable copy number, but it does not replace process control for donor biology, culture-induced drift, or post-thaw function.

## 4. Safety Considerations Following Lentiviral Transduction

Lentiviral vectors are efficient tools for stable modification of dividing and non-dividing cells, including MSCs [99,100]. Third-generation systems separate vector functions across multiple plasmids and remove accessory genes, reducing the probability of replication-competent virus formation [101,102]. Nevertheless, integration is not random: lentiviral vectors preferentially insert within transcriptionally active genes and regulatory regions [103]. The relevant safety question is therefore not whether integration occurs, but whether vector design, copy number, insertion pattern, and cell expansion create a clinically meaningful clonal risk.

### 4.1. Molecular Risks of Transduction

Self-inactivating (SIN) LTRs reduce enhancer and promoter activity from the integrated vector ends but do not eliminate insertional mutagenesis [104]. Proto-oncogene activation, tumor-suppressor disruption, and long-range regulatory effects remain possible, particularly with strong internal promoters or high vector copy number [104,105,106]. Vector copy number, integration-site analysis, growth kinetics, and evidence of clonal dominance should therefore be considered together rather than interpreted as independent release results.

Long-term clonal data for LV-modified MSCs remain limited. Barcode studies show that culture expansion itself can generate marked clonal selection [107]. In a VEGF-overexpressing MSC program, the karyotype remained stable, vector copy number was approximately 1–2 copies per cell, and no dominant clonality or tumorigenic transformation was detected during up to 4.5 months of in vivo follow-up, including conditions using an MOI up to 20 [108]. These data are reassuring for that product and observation period but do not establish multi-year safety across constructs.

### 4.2. Immunological and Cellular Risks

In several models, lentiviral transduction preserved MSC immunophenotype and differentiation and did not measurably increase alloimmune activation [100,109]. Outcomes nevertheless depend on the cargo, promoter, vector copy number, donor, and manufacturing conditions. A transgene can create a neoantigen, alter antigen-presentation pathways, or change the secretome, while inflammatory priming can simultaneously increase immunoregulatory mediators and HLA expression [57,60]. Donor-specific antibodies and immune recognition should therefore be monitored according to dosing schedule and clinical context [58].

Cellular effects may result from the transduction process rather than vector integration alone. Polybrene can suppress MSC proliferation after exposure [110], and selection or prolonged expansion can enrich atypical subpopulations. Conversely, an intended cargo such as CXCR4 can increase migration and alter biodistribution [71]. Process controls should distinguish vector-related effects, cargo-related pharmacology, and reagent toxicity and should assess migration, differentiation, senescence, and potency against the unmodified parental bank.

## 5. Current and Emerging Strategies for Enhancing Safety

Biosafety is determined by the complete product design rather than by a single platform label. SIN-LV architecture, moderate or regulated promoters, vector-copy-number limits, suicide switches, and in vivo tracking can reduce specific risks but cannot make a semirandomly integrating product risk-free. Targeted genome engineering replaces some of these uncertainties with a different set of risks—editing efficiency, off-target activity, double-strand-break-associated damage, and more complex GMP analytics—which are examined in Section 5.3.

### 5.1. Preclinical Data on Safety and Efficacy

Deletion of the U3 region from the 3′ LTR established the SIN configuration by abolishing LTR promoter activity while retaining vector production and transgene expression [111,112]. Regulated or tissue-responsive promoters can further limit the duration or location of expression [113]. Non-invasive imaging and molecular retrieval of integration sites provide complementary tools for detecting altered biodistribution or emerging clones during preclinical follow-up [114,115]. These measures reduce uncertainty but must be validated for the specific MSC source, cargo, route, and dose.

In rat BM-MSCs, second- or third-generation SIN-HIV-1 vectors preserved morphology, viability, adhesion, differentiation, and transgene expression after cryopreservation. In an in vitro ischemia model, viability of unmodified cells fell to approximately 20% by 96 h, whereas HSP70 expression significantly improved survival and reduced apoptotic nuclei and caspase-3 activity (*p* < 0.05–0.001) [100]. This study demonstrates cargo-dependent benefit under defined stress conditions; it does not by itself exclude vector-related effects in other products.

Human MSCs expressing wild-type or A168H-mutant herpes simplex virus thymidine kinase (HSV-TK) retained proliferation, immunophenotype, and differentiation. Ganciclovir selectively eliminated the modified cells, providing a pharmacologically triggered safety switch [116]. The study did not report vector copy number or integration-site maps, illustrating that a suicide system complements rather than replaces genomic characterization.

BM-MSCs expressing soluble TRAIL and/or IL-12 reduced tumor growth and mass in a murine lymphoma model. IL-12 alone and the IL-12+sTRAIL combination increased survival to as much as 60% at day 50 (*p* < 0.0001), with no reported short-term clinical toxicity and preserved MSC phenotype [117]. Longer observation and integration-site analysis would be required to evaluate delayed vector-related risk.

MSCs engineered to express IL-4, IL-10, and IL-13 retained their typical immunophenotype, karyotype, cell-cycle profile, and controlled proliferation in a diabetic-wound program. The cells localized mainly to wound sites and were not detected in lung, liver, or spleen at day 14. Wound closure exceeded 96% by day 14 and was accompanied by greater CD31+ vessel density and lower inflammatory infiltration [118]. Table 2 summarizes these preclinical examples and their product-specific limitations.

### 5.2. Clinical Data on Lentivirally Modified MSCs

Most cytokine- or death-ligand-expressing LV-MSC products remain preclinical. Published human experience is concentrated in HSV-TK suicide-gene programs for recurrent glioblastoma, so clinical maturity should not be generalized to all cargos, MSC sources, or routes of administration.

In a first-in-human, dose-escalation phase I study of allogeneic adipose-derived HSV-TK-MSCs for recurrent glioblastoma, no dose-limiting toxicity, replication-competent lentivirus, vector-associated toxicity, or immunopathological reaction was detected during follow-up of up to 18 months. Partial MRI regression occurred in 8 of 12 patients; median overall survival was 16 months and median progression-free survival was 11 months, compared with a historical overall survival of approximately 5 months [119]. The uncontrolled design and small sample prevent causal efficacy conclusions but provide early product-specific safety evidence.

A separate case report used autologous BM-MSCs carrying HSV-TK, administered intralesionally with systemic ganciclovir. The product met sterility, viability, mycoplasma, and replication-competent lentivirus criteria; no serious treatment-related adverse event was reported. Treated lesions showed slower growth and lower MRI activity than untreated lesions; progression-free survival was 293 days and survival after therapy was 513 days [120]. Table 3 summarizes the available clinical reports. In contrast, the genome-edited MSC studies discussed below remain in vitro or preclinical and do not yet provide a published human safety dataset.

### 5.3. Emerging Genome Engineering Approaches for Molecularly Defined MSC Products

Beyond introducing therapeutic transgenes, genome engineering can also reduce product variability and improve manufacturing consistency. Establishment of deeply characterized donor-derived master cell banks, predefined genomic integration sites, and targeted modification of immune-related loci such as B2M or CIITA may facilitate the development of more standardized allogeneic MSC products while reducing batch-to-batch variability.

#### 5.3.1. Lentiviral Transduction Versus Targeted Genome Engineering

Lentiviral transduction and targeted genome engineering solve different manufacturing problems. SIN-LV transduction is efficient, scalable, and clinically familiar, but it produces a distribution of insertion sites and vector copy numbers. Targeted editing can create a sequence-defined genotype and, for knock-in products, a fixed integration locus; however, precise insertion is usually less efficient than gene disruption and requires more extensive analytical control. The relevant comparison is therefore not “old” versus “new” technology, but a mature platform with residual integration uncertainty versus a less mature platform with potentially greater molecular definition and a distinct genotoxicity profile. Table 4 summarizes this trade-off using data from primary MSC studies.

#### 5.3.2. Safe-Harbor Loci and Hypoimmunogenic MSCs

A “safe harbor” is not universally safe; suitability depends on cell type, cassette size and promoter, monoallelic versus biallelic disruption, local chromatin, and the duration of observation. AAVS1 lies in the first intron of PPP1R12C on chromosome 19 and is widely used because it is accessible and supports stable expression. Its limitations are disruption of one PPP1R12C allele and context-dependent expression, so targeted clones still require phenotypic and genomic validation [123,126,127]. CCR5 lies within the coding sequence of a functional chemokine receptor and HIV co-receptor on chromosome 3. It can be useful when CCR5 disruption is acceptable or therapeutically desired, but it is not biologically neutral and should not be treated as interchangeable with AAVS1 [126,127]. CLYBL is an intronic locus on chromosome 13. In human iPSC and neural stem-cell models, CLYBL targeting achieved approximately 38–58% integration and 5–10-fold higher expression than AAVS1, with expression maintained during differentiation [128]. These data make CLYBL attractive, but they are not MSC-specific and require confirmation in the intended MSC source and manufacturing process.

Gene disruption can be substantially more efficient than precise knock-in. In primary human MSCs, tube electroporation produced B2M editing of 37.3% with Cas9 RNP alone and 80.2% when an ssODN was included for the best-performing guide; a second guide increased from 22.4% to 49.6% [124]. An optimized Cas9-RNP protocol subsequently achieved 85.1% B2M editing with viability above 90% [125]. In allogeneic activated-T-cell co-culture, B2M-knockout MSC survival was more than 2.4-fold higher than that of mock-edited cells, and CD8+ T-cell proliferation fell to below 35% of the mock condition [125]. These results provide a quantitative example of how a universal-cell design can reduce HLA class I-dependent recognition.

CIITA targeting addresses inducible HLA class II, but the most quantitative MSC-family comparison currently uses lentiviral shRNA knockdown rather than complete knockout. In IFN-γ-licensed human dental-pulp stem cells co-cultured with allogeneic PBMCs, cytotoxicity was 58.3 ± 7.2% in controls versus 12.0 ± 1.7% after B2M knockdown and 13.3 ± 0.6% after CIITA knockdown—relative reductions of approximately 79.4% and 77.2%, respectively. Activated T-cell proliferation decreased from 59.1 ± 5.4% to 30.5 ± 4.4% and 30.7 ± 4.7%, corresponding to reductions of approximately 48% [129]. The in vitro model and knockdown design limit direct extrapolation to a clinical knockout product, but the data define the expected direction and magnitude of reduced alloimmune recognition.

B2M loss alone can create an NK-cell “missing-self” signal. In umbilical-cord-derived MSCs, B2M knockout reduced allogeneic T-cell recognition but increased vulnerability to NK cells; inserting a B2M–HLA-G fusion protected against both T- and NK-cell responses while preserving MSC phenotype, trilineage differentiation, and immunosuppressive activity [130]. Thus, B2M knockout does not by itself create a universally immune-evasive product. A clinically credible design must balance T-cell evasion with NK-cell inhibition and must test infection and tumor-surveillance consequences. Operationally, hypoimmunogenic editing can reduce one important component of donor heterogeneity by allowing repeated manufacture from a single characterized donor/master bank rather than repeatedly matching or changing donors. It does not remove intrinsic donor-to-donor differences in growth, secretome, senescence, or tissue-source biology, as described in Section 2.

#### 5.3.3. Translational Limitations and GMP Implications

The principal technical limitation is the gap between efficient gene disruption and efficient precise integration in primary MSCs. The 85.1% B2M disruption obtained with RNP delivery [125] contrasts with the 7.03% AAVS1 knock-in reported in primary BM-MSCs [123]. Electroporation can reduce viability, accelerate senescence, or alter potency; low-frequency knock-in may require sorting, drug selection, or clonal expansion, each of which creates a population bottleneck and can amplify rare genomic or phenotypic abnormalities. Apparent bulk editing efficiency also does not establish biallelic status, uniformity, or absence of unedited cells.

CRISPR/Cas9 double-strand breaks can generate off-target variants, large deletions, complex on-target rearrangements, and translocations [121]. Base editors and prime editors avoid programmed double-strand breaks and can install selected nucleotide changes [131,132], but they introduce other constraints, including bystander edits, editor-dependent off-target activity, larger delivery cargos, variable efficiency in primary MSCs, and limited experience with large therapeutic cassettes. These technologies should therefore be viewed as complementary tools, not as inherently risk-free replacements for nuclease editing.

GMP implementation is substantially more complex than a research-scale edit. It requires qualified or GMP-grade nuclease, guide RNA, donor template or viral donor, a closed delivery process, controlled recovery and expansion, and validated assays for on-target structure, zygosity, copy number, unintended donor integration, off-target events, large rearrangements, residual editor components, identity, potency, sterility, and clonal composition. Deep sequencing and long-read methods increase analytical power but also cost, turnaround time, validation burden, and the challenge of setting clinically meaningful acceptance thresholds. Long-term tumorigenicity, persistence, immunogenicity, and reproductive or tissue-distribution risks remain insufficiently characterized for edited MSCs.

Despite these limitations, targeted integration directly addresses the reproducibility problem identified earlier in this review: a validated safe-harbor cassette can replace a variable distribution of lentiviral insertion sites and vector copy numbers with a predefined molecular structure. Likewise, a balanced hypoimmunogenic design can reduce HLA-dependent donor–recipient variability and support an off-the-shelf product from one master bank. The translational goal is therefore not maximal editing efficiency alone but a molecularly defined therapy whose genotype, expression level, potency, cellular composition, and residual risk can be measured and reproduced across GMP batches.

## 6. Conclusions

Genetically modified MSCs can combine tissue-directed trafficking and immunoregulation with delivery of a defined therapeutic cargo. Their clinical development is nevertheless constrained by donor and source heterogeneity, phenotypic drift, variable biodistribution, and platform-specific genomic risk. SIN-LV transduction remains the more mature route to efficient, durable expression and has limited early clinical evidence in HSV-TK MSC programs. Targeted editing offers a path to fixed loci, copy numbers, and immune-evasive genotypes, but precise knock-in efficiency, off-target and on-target rearrangements, manufacturing cost, and the absence of long-term human safety data remain major barriers.

Product quality must be controlled across donor selection, isolation, expansion, engineering, banking, cryopreservation, post-thaw recovery, and administration. Release testing should link identity and genotype to a mechanism-relevant potency assay and should include platform-specific genomic and process controls. Safe-harbor integration can reduce engineering-related batch variability by fixing the locus and copy number of a therapeutic cassette. B2M/CIITA-directed hypoimmunogenic designs can reduce HLA-dependent donor–recipient variability and enable manufacture from one characterized master bank, although baseline donor potency and NK-cell recognition still require control.

The central transition is therefore from empirically optimized MSC preparations toward molecularly defined cellular medicines. Progress will depend on choosing the engineering platform according to the intended mechanism of action and risk tolerance, integrating molecular design with risk-based quality management, and demonstrating scalable GMP manufacture and long-term safety. A product should be considered “defined” only when its genotype, expression, cellular composition, potency, biodistribution, and genomic risk are reproducible and clinically interpretable.

## Figures and Tables

**Table 1:** Representative phenotypic and isolation characteristics of MSCs from major tissue sources.

MSC Source	Representative Additional Markers	Biological/Phenotypic Comments	Isolation Characteristics
BM-MSCs	CD146^+^, CD106^+^, CD271^+^; variable SSEA-4 and CD133	Perivascular-associated populations; strong osteogenic potential; donor age can markedly affect expansion and potency.	Invasive iliac-crest or sternal aspiration; MSCs comprise approximately 0.001–0.01% of marrow mononuclear cells.
AD-MSCs	CD73^+^, CD90^+^, CD105^+^, CD29^+^, CD44^+^, CD166^+^; generally lower CD146 than BM-MSCs	High initial yield and proliferative capacity; tissue factor expression should be considered for intravascular use.	Lipoaspiration is less invasive; the stromal vascular fraction contains substantially more stromal/progenitor cells than bone marrow.
WJ-MSCs	CD56^+^, CD105^+^; intermediate CD146; variable SSEA-4	Perinatal phenotype with high expansion capacity and suitability for allogeneic banking.	Non-invasive postnatal collection from Wharton’s jelly; abundant source with no additional donor risk.

**Table 2:** Selected preclinical studies of lentivirally modified MSCs.

Cell-Based Product/Model	Safety Observations	Therapeutic Effect	Ref.
Rat BM-MSCs expressing HSP70; in vitro ischemia	Morphology, viability, adhesion, differentiation, and post-thaw expression were preserved.	Unmodified viability fell to ~20% at 96 h; HSP70 reduced apoptosis and improved survival.	[100]
Human HSV-TK-MSCs; suicide-switch model	Phenotype and differentiation were retained; ganciclovir selectively eliminated modified cells. VCN/integration sites were not reported.	Conditional ablation provided control of unwanted persistence.	[116]
BM-MSCs expressing sTRAIL and/or IL-12; murine lymphoma	No reported short-term clinical toxicity; MSC phenotype was preserved.	Reduced tumor growth; survival reached up to 60% at day 50.	[117]
MSCs expressing IL-4/IL-10/IL-13; diabetic-wound model	No karyotypic, cell-cycle, or uncontrolled-growth abnormalities; restricted day-14 biodistribution.	>96% wound closure at day 14 with increased vascular density.	[118]

**Table 3:** Published clinical experience with lentivirally modified MSCs.

Product/Study	Safety Observations	Clinical Observations	Ref.
Allogeneic AD-MSCs expressing HSV-TK; 12-patient phase I glioblastoma study	No dose-limiting or vector-associated toxicity; no RCL; follow-up to 18 months.	MRI partial regression in 8/12; median OS 16 months and PFS 11 months.	[119]
Autologous BM-MSCs expressing HSV-TK; single-patient case report	No serious treatment-related event; sterility, viability, mycoplasma, and RCL criteria met.	Treated lesions progressed more slowly; PFS 293 days and post-treatment survival 513 days.	[120]

**Table 4:** Direct comparison of SIN-LV transduction and targeted genome engineering in MSCs.

Criterion	SIN-LV Transduction	Targeted Editing/Safe-Harbor Knock-in
Integration pattern	Semirandom integration enriched in transcriptionally active chromatin; each lot contains a distribution of sites and vector copy numbers.	Sequence-directed cleavage; a validated knock-in can fix locus and copy number, but allelic mixtures and unintended donor integration must be excluded.
Insertional/genomic risk	Residual insertional mutagenesis and clonal-selection risk despite SIN LTRs; risk rises with strong promoters and higher vector copy number [104,105,106,107,108].	Avoids semirandom vector integration, but off-target cleavage, large on-target deletions/rearrangements, translocations, and random donor capture remain possible [121].
Efficiency in primary MSCs	Optimized transduction was ~50%, increased to 68% by reducing volume and to 79% with FGF-2; expression persisted for 6 weeks [122].	AAVS1 knock-in yielded 7.03% GFP^+^ BM-MSCs [123]. B2M disruption reached 37.3–80.2% with Cas9/ssODN electroporation [124] and 85.1% with Cas9 RNP at >90% viability [125].
Expression stability	Generally durable after integration, but magnitude can vary with insertion site, copy number, promoter choice, and silencing.	Potentially more predictable at a validated locus. Six AAVS1-targeted clones secreted 8.7–9.3 ng hFIX/10^6 cells/24 h with little inter-clone variation [123].
Batch-to-batch reproducibility	Transduction rate and vector copy number vary with donor, passage, MOI, enhancer, and vector lot; VCN and integration-site controls are needed.	A fixed locus and copy number can reduce engineering-related variability and support a genotype-defined master bank; donor-dependent baseline potency still requires control.
GMP complexity and cost	Established vector production and release workflows, but RCL, VCN, residual components, and risk-based integration-site testing add cost.	GMP nuclease/guide/donor manufacture, closed electroporation, enrichment or cloning, expansion after a bottleneck, and deep on/off-target analytics generally increase cost and release time.
Clinical maturity	More mature: published LV-HSV-TK MSC clinical experience includes follow-up to 18 months [119,120], in addition to broader clinical LV experience in other cell types.	The cited MSC studies are in vitro or preclinical. Long-term human safety, persistence, immunogenicity, and clonal behavior remain uncharacterized.

## Data Availability

No new data were created or analyzed in this study. Data sharing is not applicable to this article.
